# A multidimensional analysis of neuropsychiatric lupus: clinical, biological and imaging insights from systematic evidence

**DOI:** 10.3389/fimmu.2026.1768131

**Published:** 2026-03-16

**Authors:** Karim Matmat, Hélène Jamann, Amin Maazouzi, Kévin Bigaut, Michel Maitre, Jérôme De Seze, Ayikoé-Guy Mensah-Nyagan, Hélène Jeltsch-David

**Affiliations:** 1 Institut National de la Santé et de la Recherche Médicale (INSERM) – Centre d’Investigation Clinique 1434, Neuroprotection and Remyelination – Centre de Recherche en Biomédecine de Strasbourg, University of Strasbourg, Strasbourg, France; 2 Strasbourg University Hospital, Strasbourg, France; 3 Neurology Department, Hautepierre Hospital, Strasbourg University Hospital, Strasbourg, France; 4 Integrative Multimodal Imaging in Healthcare (IMIS), Laboratory of Engineering, Informatics and Imaging (ICube), Centre National de la Recherche Scientifique (CNRS), Unité Mixte de Recherche 7357 (UMR 7357), Université de Strasbourg, Strasbourg, France

**Keywords:** autoimmune diseases, biomarkers, neuroinflammation, neuropsychiatric systemic lupus erythematosus (NPSLE), systematic review

## Abstract

**Objective:**

Neuropsychiatric systemic lupus erythematosus (NPSLE) is among the most challenging manifestations of systemic lupus erythematosus (SLE), affecting the central and peripheral nervous systems with diverse symptoms. Despite its prevalence, the diagnosis and management of NPSLE remain complex due to the heterogeneity of clinical presentations and the lack of specific biomarkers. This study aims to synthesize data from case reports to better understand the clinical, biological, and imaging features of NPSLE.

**Methods:**

A systematic review with integrated descriptive and exploratory quantitative analyses of 120 published case reports was conducted, integrating demographic, clinical, biological, and imaging data, as well as therapeutic approaches. Subgroup analyses stratified by age and sex were performed to identify descriptive patterns of presentation and outcomes.

**Results:**

Age- and sex-related patterns were observed in disease presentations. Patients presenting at younger ages more frequently exhibited renal and cutaneous involvement, whereas those presenting in adulthood or at older ages more often displayed neurological and neuropsychiatric manifestations, including motor dysfunction, cognitive impairment, and mood disorders. Male patients exhibited higher rates of severe renal involvement, whereas neuropsychiatric symptoms were more frequently reported, particularly among female cases. Neuroimaging abnormalities were identified in over 80% of patients in whom imaging was performed, with common findings including white matter lesions, cortical atrophy, and focal gray matter involvement. Despite the widespread use of corticosteroids and immunosuppressants, treatment gaps persisted, particularly in the management of neuropsychiatric symptoms.

**Conclusion:**

This review highlights the need for the development of biomarker-driven diagnostic tools and targeted therapies to address the unmet needs of NPSLE patients, while advances in imaging and biologics hold promises for improving patient outcomes. These findings should be interpreted as hypothesis-generating, given the descriptive nature of case-report–based evidence.

## Introduction

Systemic lupus erythematosus (SLE) is a heterogeneous autoimmune disease characterized by chronic inflammation and multiorgan involvement. Often described as “the disease with a thousand faces”, SLE presents with striking clinical variability that complicates timely diagnosis and therapeutic decision-making (1). Among its most severe manifestations is neuropsychiatric lupus (NPSLE), a broad spectrum of neurological and psychiatric syndromes affecting both the central and peripheral nervous systems. Up to 75% of patients develop neuropsychiatric symptoms during the disease course, ranging from subtle cognitive or mood disturbances to life-threatening complications such as psychosis, cerebrovascular events, or seizures (2). Despite decades of research, the epidemiology, mechanisms, and optimal management of NPSLE remain poorly defined, particularly across diverse demographic subgroups.

The pathogenesis of NPSLE is multifactorial, involving systemic autoimmunity, blood–brain barrier (BBB) dysfunction, neuroinflammation, and microvascular injury (3, 4). Multiple mechanisms have been proposed, autoantibody-mediated neuronal or endothelial injury, cytokine-driven inflammation, microglial activation, and direct neurotoxicity (5). Biomarkers such as anti-ribosomal P antibodies, anti-NMDA receptor antibodies, antiphospholipid antibodies, and anti-aquaporin-4 antibodies have been associated with specific neuropsychiatric phenotypes (6, 7). However, none offer sufficiently robust diagnostic or prognostic performance for routine clinical use. Diagnostic uncertainty is exacerbated by the overlap of NPSLE with other neurological conditions, including multiple sclerosis and primary psychiatric disorders, often relegating diagnosis to exclusion criteria rather than positive biological indicators (8). This highlights a critical unmet need for reliable biomarkers and integrative approaches capable of capturing the biological and phenotypic heterogeneity of NPSLE.

Management is equally challenging. No evidence-based, syndrome-specific therapeutic guidelines exist, and current treatments—corticosteroids, immunosuppressants, anticoagulants, or antipsychotics—are largely empirical, guided by presumed mechanisms rather than validated clinical algorithms (9, 10). For milder or overlapping presentations, the indication for immunosuppression remains debated, and the adverse effects of aggressive therapy further complicate decision-making.

Neuroimaging techniques such as magnetic resonance imaging (MRI) have identified structural and functional abnormalities in NPSLE, including white matter lesions, cerebral atrophy, and perfusion deficits (11–13). Yet imaging findings are often nonspecific and rarely sufficient to distinguish NPSLE from other neuroinflammatory or neurovascular disorders (14). Emerging evidence suggests that symptom patterns may correlate with specific biological markers, imaging profiles, or demographic factors (15, 16), but these associations remain fragmented across the literature and difficult to interpret clinically.

Given these persistent gaps, a comprehensive and integrated synthesis is needed. The present systematic review aims to characterize the spectrum of neuropsychiatric manifestations in SLE across diverse patient populations; examine relationships between clinical phenotypes, demographic variables, biomarkers, and neuroimaging findings; and assess current treatment strategies and their limitations. By consolidating available evidence and highlighting recurring mechanistic and clinical patterns, this review seeks to provide an updated framework for understanding NPSLE, facilitate earlier and more accurate diagnosis, and support the development of targeted, personalized therapeutic approaches.

## Methods

### Data sources and search strategy

This systematic review was conducted in accordance with the PRISMA (Preferred Reporting Items for Systematic Reviews and Meta-Analyses) guidelines. A comprehensive literature search was performed using MEDLINE (via PubMed) and the Cochrane Library. The literature search covered publications from January 1995 to August 2024. The following search strategy was applied: (“systemic lupus erythematosus”[MeSH Terms] OR lupus OR SLE) AND (NPSLE OR neurolupus OR “neuropsychiatric lupus” OR “neuropsychiatric involvement” OR “lupus cerebritis”) AND (“case reports”[Publication Type] OR “case report” OR “case series”).

Reference lists of relevant papers were manually screened to identify additional eligible reports.

### Study selection

Studies were included if they reported original clinical data describing neurological and/or neuropsychiatric manifestations in patients with a diagnosis of SLE attributed to NPSLE. Exclusion criteria were: non–peer-reviewed material (gray literature, theses, dissertations), conference abstracts or inaccessible full texts, interventional clinical trials, studies lacking extractable clinical or biological data, articles not written in English.

Two reviewers (K.M. and H.J.) independently screened titles/abstracts and evaluated full texts. Discrepancies were resolved by consensus.

### PICOS framework

This systematic review was conducted according to the PICOS framework:

- Population: Patients diagnosed with systemic lupus erythematosus presenting neuropsychiatric manifestations attributed to NPSLE.
- Intervention: Clinical, biological, and imaging evaluations reported in published case reports.
- Comparator: Not applicable, given the descriptive and observational nature of the included studies.
- Outcomes: Neurological and neuropsychiatric manifestations, biological and immunological parameters, neuroimaging findings, treatments, and clinical outcomes.
- Study design: Published peer-reviewed case reports and case series.

### Data collection and extraction

Data were extracted using a predefined, structured template encompassing nine domains: (1) Study characteristics (first author, year of publication, DOI). (2) Demographics (sex, age at first clinical diagnosis, age at last follow-up when available). Age at first diagnosis was defined as the earliest documented age at which a diagnosis related to SLE was reported, either at SLE diagnosis or, when no prior SLE diagnosis was described, at inaugural NPSLE presentation. (3) General SLE features (cutaneous, articular, renal involvement; treatments received). (4) Neurological symptoms coded as binary variables (motor/coordination disorders, sensory/perceptual deficits, seizures/convulsions, consciousness disorders). (5) Neuropsychiatric symptoms coded as binary variables (cognitive impairment, mood/affective disorders, and behavioral or psychotic disturbances). Neurological and neuropsychiatric symptom groupings were defined as aggregated functional categories for harmonization across heterogeneous case reports, and were aligned with the 1999 American College of Rheumatology nomenclature for neuropsychiatric lupus syndromes; for example, sensory or perceptual deficits include peripheral nervous system manifestations, such as mono- or polyneuropathy, disorders of consciousness correspond to acute confusional states, and neuropsychiatric disturbances encompass ACR-defined syndromes such as cognitive dysfunction, mood disorders, and psychosis. (6) Neuroimaging findings (cerebral MRI, CT, angiography). (7) Biological parameters (*e.g.;* hematology, biochemistry, autoantibodies, and cerebrospinal fluid (CSF) analysis). (8) Treatment information (*e.g.;* immunosuppressive, anticoagulant, antipsychotic, and adjunctive therapies). (9) Clinical course and outcomes (symptom resolution, persistence, or death). Data extraction was performed independently by both reviewers, with disagreements resolved through discussion.

### Quality assessment and risk of bias

Given the nature of the included studies (case reports and small case series), methodological quality was assessed using an adapted descriptive framework focusing on the completeness of clinical description, diagnostic attribution to NPSLE, reporting of biological and imaging findings, and clarity of clinical outcomes. Studies lacking sufficient clinical detail or clear attribution of neuropsychiatric manifestations to SLE were excluded during the screening process. Given the exploratory nature of case-report synthesis, no formal risk-of-bias scoring tool was applied.

### Main outcome measures

The primary objective was to characterize the clinical, neurological, neuropsychiatric, biological, and imaging features of NPSLE. Secondary objectives included exploring potential associations between demographics, symptom clusters, biomarkers, imaging abnormalities, treatments, and clinical outcomes. Exploratory multivariate modelling and visualization tools were used to support interpretation of potential mechanistic pathways.

### Quantification and statistical analysis

#### Descriptive analysis

Continuous variables were summarized as medians with interquartile ranges (IQR). Categorical variables were reported as counts and percentages. Sex-related differences in symptom prevalence were assessed using Chi² tests or Fisher’s exact test. Associations with age at disease diagnosis (juvenile, adult, elderly) were evaluated using the Cochran–Armitage trend test (two-sided).

#### Data processing and imputation

Missing biological data were handled using multiple imputation by chained equations (MICE) with a random forest method. Prior to imputation, missingness patterns were explored by examining associations between missingness indicators and observed variables, indicating structured missingness inconsistent with a completely random mechanism. Accordingly, imputation was conducted for exploratory purposes without explicitly assuming a specific missingness mechanism. Five imputed datasets were generated over 50 iterations. Convergence and imputation quality were assessed using visual diagnostics (*e.g.*, density plots comparing observed and imputed distributions). Analyses were performed in R (v2024.09.0) using the mice package. Of note, imputed values were not used to estimate biomarker prevalence or absolute frequencies but solely to support exploratory multivariate structure analysis.

### Exploratory multivariate analysis and variable clustering

To identify multivariate patterns across biological markers, a principal component analysis (PCA) was conducted on the imputed datasets as an unsupervised dimensionality-reduction approach. The number of components and associated cluster structure was guided by the elbow method, based on inspection of explained variance across increasing component numbers. PCA computation and cluster visualization were performed using the *FactoMineR* and *factoextra* R packages.

For each patient, biological and biochemical parameters were first dichotomized as normal or abnormal based on the reference ranges or qualitative interpretations reported in the original case reports. Biomarkers were subsequently grouped into biologically coherent clusters using the exploratory multivariate framework.

For each biological cluster, an activity score was defined as the proportion of constituent biomarkers classified as abnormal for a given patient. A cluster was considered predominantly abnormal when more than 50% of its biomarkers were reported as abnormal. These cluster activity scores were used as predictors in logistic regression models to assess associations with neurological and neuropsychiatric symptom categories. Neuroimaging abnormalities and treatment variables were analyzed using the original (non-imputed) dataset. Logistic regression models assessed associations with clinical outcomes, including symptom improvement or persistence. Odds ratios (ORs) and 95% confidence intervals (CIs) were calculated. False discovery rate (FDR) correction (Benjamini–Hochberg) was applied to adjust for multiple comparisons. Variables with FDR < 0.05 were considered statistically significant. Results were visualized using *forest plots* generated with the *forestplot* R package.

## Results

### Literature review

A systematic search following PRISMA guidelines (**Supplementary Figure 1**) initially identified 312 publications. After removal of duplicates and application of inclusion and exclusion criteria, 92 studies met eligibility requirements and were included in the final analysis (17–108). Together, these studies reported 120 individual NPSLE cases, providing detailed information on demographics, clinical features, biomarkers, neuroimaging, treatments, and outcomes. A comprehensive summary of epidemiological and clinical characteristics is provided in **Supplementary Table 1**.

### Description of the whole population of 120 NPSLE patients

The cohort comprised 120 patients with NPSLE, with a marked female predominance (101 females, 84.2%) compared with males (19 males, 15.8%). The median age at first documented disease diagnosis was 22.5 years (IQR 16–36.2), and the median age at the last recorded evaluation was 28.5 years (IQR 20–41.2), indicating that most patients were young adults at the time of clinical documentation and follow-up. The age at diagnosis could correspond either to SLE diagnosis or to inaugural NPSLE, depending on the information reported in the original case descriptions. Due to heterogeneous reporting, the age at biological SLE diagnosis and the time interval between SLE diagnosis and NPSLE involvement could not be summarized quantitatively across the cohort.

Patients were categorized into three age groups based on age at first documented diagnosis: juvenile (<16 years), adult (16–50 years), and elderly (>50 years) (109, 110). Adult cases were the most frequent (82/120, 68.3%), followed by juvenile (28/120, 23.3%) and elderly cases (10/120, 8.3%). A comparable distribution was observed across sexes. Among women, 69.3% (70/101) were diagnosed during adulthood, 21.8% (22/101) during childhood or adolescence, and 8.9% (9/101) at older age. Male patients showed a similar pattern (63.2% adult, 31.6% juvenile, 5.3% elderly).

### Phenotypic landscape of NPSLE patients according to age and sex

The analysis of symptom prevalence across age-of-onset focused on six major clinical domains relevant to SLE and NPSLE: renal involvement, dermatological manifestations, arthritis-related symptoms, neurological features, and neuropsychiatric presentations, and hematologic involvement (**Supplementary Tables 1** and **2**). Overall, renal symptoms were more frequent at both extremes of age, whereas dermatological manifestations predominated in the juvenile group. Skin involvement was observed in 64.3% (18/28) of juvenile patients, compared with 42.7% (35/82) of adult and 50% (5/10) of elderly patients, without evidence of a non-significant age-related trend (*P* = 0.148). In contrast, arthritis and related joint symptoms were more frequent in adult (51.2%, 42/82) and elderly patients (60%, 6/10) than in juvenile cases (28.6%, 8/28), showing a significant age-related increase (*P* = 0.031).

Neurological symptoms, including motor and vesicosphincterian deficits, coordination or sensory disturbances and seizures, were highly prevalent across all age groups, with an age-related increase: neurological involvement was reported in 78.6% (22/28) of juvenile cases, 90.2% (74/82) of adult cases, and 100% (10/10) of elderly patients (*P* = 0.041). Neuropsychiatric manifestations displayed a similar pattern, being most frequent in elderly patients (80%, 8/10), followed by adult patients (70.7%, 58/82), and in juvenile (50%, 14/28), indicating a significant age-related trend (*P* = 0.032).

Sex-based comparisons revealed additional differences. Renal involvement was more frequent in male patients (52.6%, 10/19) than in female patients (33.7%, 34/101), although this difference did not reach statistical significance (*P* = 0.13). Conversely, dermatological manifestations were more common among women, affecting 51.5% (52/101) compared with 31.6% (6/19) of men, but this difference was not statistically significant at the cohort level (*P* = 0.14). Arthritis-related symptoms showed no meaningful sex difference, occurring in 42.1% of males and 47.5% of females (*P* = 0.80). Finally, neurological and neuropsychiatric symptoms affected both sexes at similar rates: neurological involvement occurred in 87.1% of female and 94.7% of male patients (*P* = 0.70), and neuropsychiatric manifestations in 67.3% of female and 63.2% of male patients (*P* = 0.79). These findings indicate that, although trends toward sex-related differences in renal and cutaneous involvement were observed, neurological and neuropsychiatric complications appear broadly comparable between males and females when considering the entire NPSLE cohort.

### NPSLE phenotypes and biological/imaging associations

#### Biological parameters

Patients with NPSLE displayed widespread abnormalities across hematological, biochemical, immunological, and inflammatory parameters, reflecting the systemic and multidimensional nature of the disease (**Supplementary Table 2**). Hematological abnormalities were particularly common. Anemia was present in 61.1% (55/90) of cases and was predominantly normocytic, with normal MCV values in 89.8% (53/59) of patients, suggesting anemia of chronic inflammation or immune-mediated hemolysis rather than nutritional deficiencies. This was supported by a weak positive correlation between hemoglobin and MCV (ρ = 0.21) (**Supplementary Figure 2**). Leukopenia was observed in 43% (40/93) of patients, and lymphopenia in 41.6% (32/77), indicating profound immune dysregulation and potentially increased infectious vulnerability. Thrombocytopenia occurred in 41.6% (32/77) of cases. Platelet levels showed weak negative correlations with C3 (ρ = -0.16) and C4 (ρ = -0.02), but a stronger positive correlation with anticardiolipin IgM (ρ = 0.34 (**Supplementary Figure 2**).

Biochemical markers underscored renal involvement as a major feature of NPSLE. Proteinuria was highly prevalent, affecting 84.8% (56/66) of patients, consistent with lupus glomerulonephritis. Elevated creatinine levels were less frequent (14.6%, 13/89) but indicated more severe renal impairment in a subset of patients. Electrolyte levels were generally preserved, with normal sodium in 85.5%, potassium in 88.1%, and chloride in 92% of patients. Systemic inflammatory markers were frequently elevated, with CRP increased in 71.8% of cases and ESR in 91.9%, indicating a substantial inflammatory burden. CRP and ESR showed a moderate correlation (ρ = 0.40)(**Supplementary Figure 2**).

Immunological parameters revealed profound autoantibody alterations characteristic of active SLE. Anti-dsDNA antibodies were detected in 84.5% (93/110) of patients and moderately correlated with both C3 (ρ = 0.32) and C4 (ρ = 0.38). Anti-SSA and anti-SSB antibodies were present in 77% and 50% of patients, respectively, and showed correlations with each other (ρ = 0.46) and with anti-RNP (ρ = 0.41), indicating shared autoimmune pathways. Anti-SSB also correlated moderately with anti-RNP (ρ = 0.30). ANA positivity was nearly universal (99.2%), although its correlation with anti-dsDNA was weak (ρ = 0.22). Antiphospholipid antibodies demonstrated strong correlations with anti-β2-glycoprotein I, including anticardiolipin IgM (ρ = 0.54), anticardiolipin IgG (ρ = 0.59), and lupus anticoagulant (ρ = 0.60) (**Supplementary Figure 2**).

Complement depletion was a frequent finding, with reduced C3 levels in 77% (77/100) and reduced C4 in 79.8% (75/94) of patients. A strong positive correlation between C3 and C4 (ρ = 0.83) reflected their parallel consumption during immune complex formation. Anti-Sm antibodies were detected in 74.5% of patients and strongly correlated with anti-RNP (ρ = 0.64)(**Supplementary Figure 2**).

Finally, cerebrospinal fluid (CSF) abnormalities were observed in 59.8% (46/77) of patients. These abnormalities showed modest correlations with anti-Sm (ρ = 0.24) and anti-RNP antibodies (ρ = 0.29) (**Supplementary Figure 2**).

#### Neuroimaging findings in patients with NPSLE

Neuroimaging abnormalities were common among patients with available data. Brain MRI was the most sensitive modality, identifying abnormalities in 81.6% (80/98) of cases, compared with 55% (22/40) for head CT and 50% (5/10) for CT angiography (**Supplementary Table 1**). Associations between imaging findings and clinical manifestations revealed additional patterns. MRI abnormalities were significantly associated with neurological symptoms (OR 2.62, 95% CI 1.37–5.18; FDR = 0.01), whereas CT (OR 1.31, 95% CI 0.58–3.56; FDR = 0.83) and CT angiography (OR 1.14, 95% CI 0.29–15.73; FDR = 0.88) showed no significant associations. For neuropsychiatric symptoms, MRI demonstrated a possible trend (OR 1.61, 95% CI 0.99–2.64; FDR = 0.08) without reaching statistical significance. CT findings (OR 1.32, 95% CI 0.77–2.39; FDR = 0.33) and CT angiography (OR 0.31, 95% CI 0.10–0.79; FDR = 0.07) did not show consistent associations.

Age-stratified analyses revealed distinct profiles. In the juvenile cases, MRI abnormalities were reported in 82.3% (14/17) of patients, compared with 75% (3/4) on CT and 50% (1/2) on CT angiography. Adult patients showed MRI abnormalities in 79.2% (57/72), CT abnormalities in 53.1% (17/32), and CT angiography abnormalities in 50% (4/8). Among elderly patients, MRI consistently identified abnormalities in all cases (100%, 9/9), while CT abnormalities were present in 50% (2/4), and no abnormalities were detected on CT angiography. Gender-stratified analyses showed similar trends. Among women, MRI abnormalities were reported in 79.8% (67/84), CT abnormalities in 60% (21/35), and CT angiography abnormalities in 50% (4/8). Male patients demonstrated even higher MRI sensitivity, with abnormalities detected in 92.9% (13/14); however, CT abnormalities were infrequent (20%, 1/5) while CT angiography abnormalities were detected in 50% (1/2).

Together, these findings highlight MRI as the most reliable imaging modality for detecting CNS abnormalities in NPSLE across age and sex groups, while CT- and CT-angiography-based evaluations show lower and more variable sensitivity.

### Biological signatures associated with clinical phenotypes

To investigate the biological determinants of symptom heterogeneity in NPSLE, principal component analysis (PCA) was applied to reduce dimensionality and group biomarkers according to shared variance. This approach identified four distinct biological clusters, each corresponding to a coherent pathophysiological domain: “*Autoimmunity and Inflammation*”, “*Kidney and Metabolic Dysfunction*”, “*Thrombosis and Antiphospholipid Syndrome*”, and “*Hematology and Immune Cells*” (**Supplementary Figure 3**). These clusters were subsequently used as explanatory variables in logistic regression models to explore their associations with major clinical symptom categories. Odds ratios (ORs) and 95% confidence intervals (CIs) were computed to quantify effect sizes.

**Figure 1:**
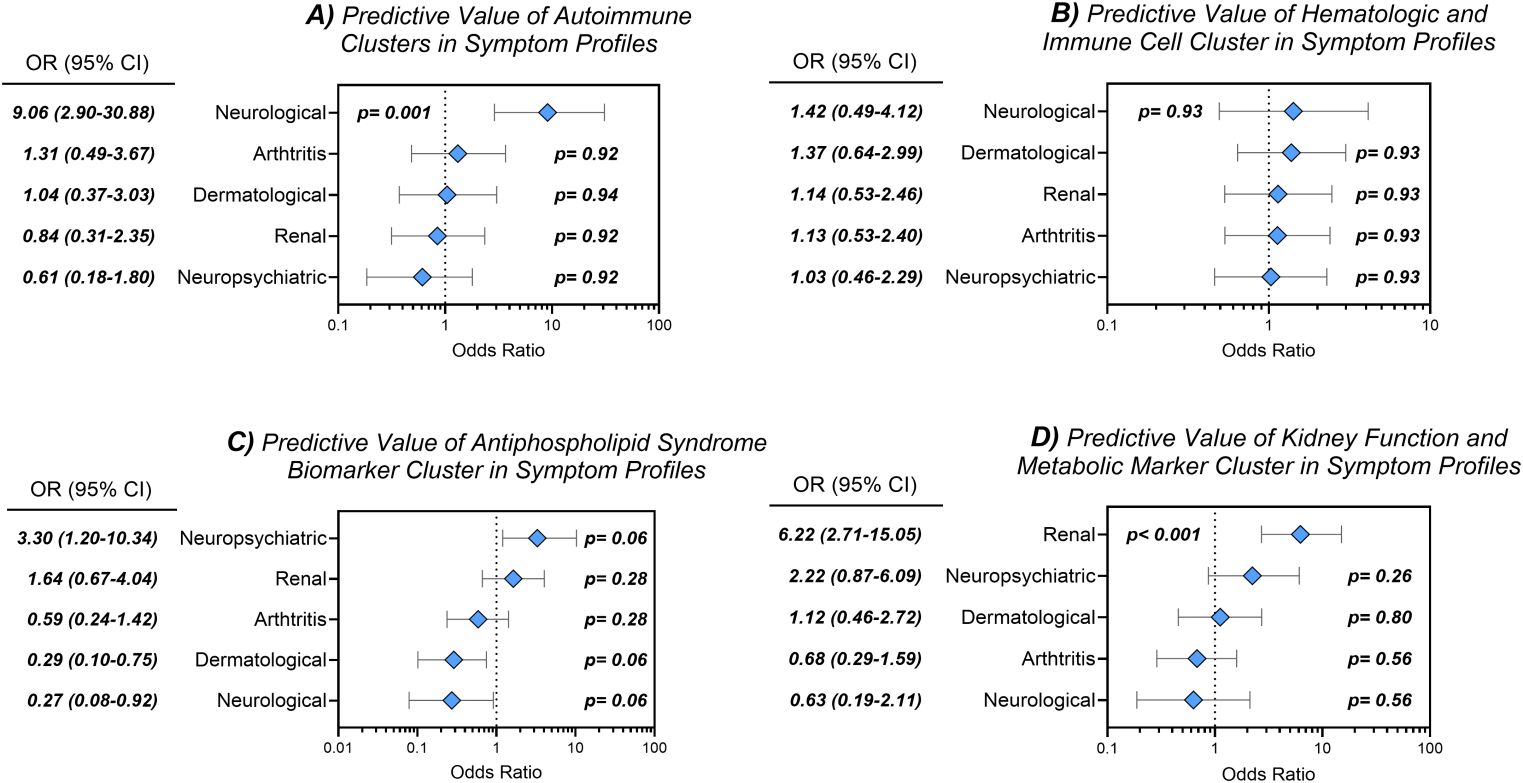
Predictive value of biomarker clusters for symptom profiles. Logistic regression analysis showing odds ratios (OR) and 95% confidence intervals (CI) for associations between biomarker clusters and symptom categories. **(A)** Autoimmunity and Inflammation cluster. **(B)** Hematology and Immune Cells cluster. **(C)** Kidney and Metabolic Dysfunction cluster. **(D)** Thrombosis and Antiphospholipid Syndrome cluster. Blue diamonds indicate OR; horizontal lines represent 95% CI.

The “*Autoimmunity and Inflammation*” cluster demonstrated a strong association with neurological symptoms (OR 9.06, 95% CI 2.90–30.88; *P* = 0.001) (**Figure 1A**). However, this cluster showed no significant associations with dermatological, renal, arthritis-related, or neuropsychiatric symptoms. The “*Hematology and Immune Cells*” cluster did not exhibit significant associations with any symptom category (**Figure 1B**).

The “*Kidney and Metabolic Dysfunction*” cluster was strongly associated with renal manifestations (OR 6.22, 95% CI 2.71–15.05; *P* < 0.001) (**Figure 1C**). No associations were observed between this cluster and neurological, neuropsychiatric, dermatological, or joint-related symptoms, underscoring its specificity for renal pathology. Finally, the “*Thrombosis and Antiphospholipid Syndrome*” cluster showed a non-significant trend toward association with neuropsychiatric symptoms (OR 3.30, 95% CI 1.20–10.34; *P* = 0.059) (**Figure 1D**). No significant associations were identified between this cluster and renal, dermatological, joint, or neurological features.

### Characterization of neurological and neuropsychiatric symptom profiles

To better capture the heterogeneity of clinical presentations in NPSLE, neurological and neuropsychiatric symptoms were categorized into distinct subgroups. Neurological symptoms were classified into three domains: (1) vesicosphincterian and coordination disorders, reflecting impairments in bladder regulation and motor coordination; (2) sensory and perceptual disorders, encompassing disturbances in sensory processing; and (3) seizures/convulsions and consciousness disorders, representing severe manifestations indicative of central nervous system dysfunction. Neuropsychiatric symptoms were grouped into (1) cognitive disorders, involving deficits in memory, attention, and executive functioning; (2) mood and affective disorders, including depressive and anxiety-related symptoms; and (3) behavioral and psychotic disorders, capturing abnormalities in behavior, thought processes, or perception.

#### Overall description

Across the cohort, neurological and neuropsychiatric symptoms displayed substantial variability in frequency (**Supplementary Table 3**). Vesicosphincterian, motor, and coordination impairments were present in 54.7% (58/106) of patients, whereas sensory and perceptual disturbances affected 49% (52/106). Seizures, convulsions, and consciousness disorders were the most prevalent neurological manifestations, reported in 59.4% (63/106). Among neuropsychiatric symptoms, cognitive impairment was particularly common, occurring in 77.5% (62/80) of individuals and representing the most frequent neuropsychiatric domain. Mood and affective disorders were documented in 57.5% (46/80) of cases, and behavioral or psychotic disturbances were observed in 46.2% (37/80).

#### Symptom analysis across age groups and sex

**Figure 2:**
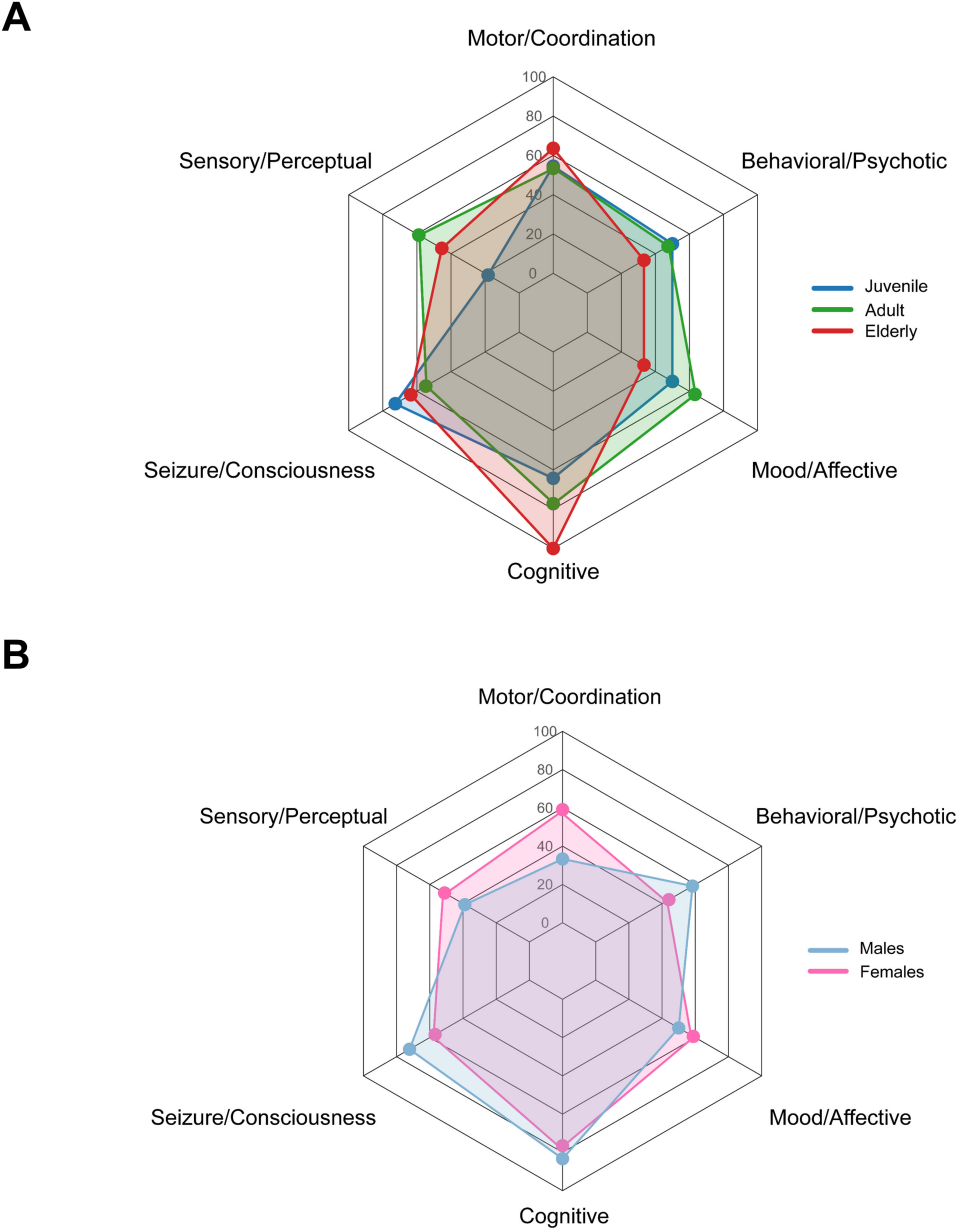
Frequency of neurological and neuropsychiatric symptoms by age at presentation and sex. **(A)** Neurological and neuropsychiatric symptom categories are shown across juvenile, adult, and elderly age-at-presentation groups. **(B)** Symptom frequencies are compared between male and female groups.

Sensory and perceptual disorders were reported in 49.1% (52/106) of the cohort. Their prevalence was lowest in juvenile patients (18.2%, 4/22) and substantially higher in adult (58.9%, 43/73) and elderly groups (45.4%, 5/11), showing a significant age-related trend (*P* = 0.023). A sex-based difference was also observed, with females more frequently affected (51.1%, 45/88) than males (38.9%, 7/18), although this difference did not reach statistical significance (*P* = 0.44) (**Figures 2A, B**).

Seizures, convulsions, and consciousness disorders represented the most common neurological manifestations overall. These symptoms were particularly frequent in juvenile patients (72.7%, 16/22), somewhat lower in adult individuals (54.8%, 40/73), and again elevated in elderly patients (63.6%, 7/11), without evidence of a significant age-related trend (*P* = 0.37). A similar pattern emerged for sex, with higher prevalence in males (72.2%, 13/18) compared with females (56.8%, 50/88), although this difference was not statistically significant (*P* = 0.30). Cognitive impairment was the most prominent neuropsychiatric feature, affecting 77.5% (62/80) of patients. Its prevalence increased markedly with age, from 64.3% (9/14) in juvenile patients to 77.2% (44/57) in adult patients and 100% (9/9) in elderly patients, indicating a significant age-related trend (*P* = 0.051). Prevalence was similarly high in both sexes, affecting 76.5% (52/68) of females and 83.3% (10/12) of males (*P* = 0.73).

Mood and affective disorders were reported in 57.5% (46/80) of patients. Prevalence showed moderate variation across age groups, being present in 50% (7/14) of juvenile patients, 63.2% (36/57) of adult, and 33.3% (3/9) of elderly patients, without a significant age-related trend (*P* = 0.63). Female patients exhibited slightly higher rates (58.8%, 40/68) than male patients (50%, 6/12), but this difference was not statistically significant (*P* = 0.57). Behavioral and psychotic disorders were the least frequent neuropsychiatric manifestations, occurring in 46.2% (37/80) of patients overall. They were reported in 50% (7/14) of juvenile cases, 47.4% (27/57) of adult and 33.3% (3/9) of elderly cases, with no significant age-related trend (*P* = 0.48). These symptoms were more common in males (58.3%, 7/12) than in females (44.1%, 30/68), although this difference did not reach statistical significance (*P* = 0.36) (**Figures 2A, B**). Together, these findings show distinct age-related gradients for specific neurological and neuropsychiatric domains, particularly sensory disturbances and cognitive impairment, while sex-related differences remain limited and do not reach statistical significance at the cohort level.

#### Symptom profiles across imaging modalities

To complement the characterization of symptom patterns, associations between neurological and neuropsychiatric manifestations and imaging modalities were assessed by calculating odds ratios (ORs), 95% confidence intervals (CIs), and FDR-adjusted P values for each symptom modality. MRI demonstrated the strongest associations. It was significantly associated with motor, vesicosphincterian, and coordination disorders (OR 2.62, 95% CI 1.37–5.18; *P* = 0.012), as well as with sensory and perceptual disturbances (OR 1.80, 95% CI 1.10–3.00; *P* = 0.045). In contrast, no significant associations emerged between MRI findings and seizures/convulsions, consciousness disorders, cognitive impairment, mood or affective disturbances, or behavioral and psychotic symptoms. Similarly, CT scans did not show significant associations with any neurological or neuropsychiatric symptom categories, and CT angiography failed to reveal meaningful associations across all evaluated symptoms. Taken together, these findings highlight MRI as the most informative imaging modality for detecting structural abnormalities linked to specific neurological deficits—particularly motor and sensory disturbances—and reinforce its central role in the diagnostic evaluation of NPSLE.

### Treatments and outcomes

The therapeutic landscape across the cohort showed a strong reliance on corticosteroids, which were administered to 81.7% (98/120) of patients. Prednisone, prednisolone, and methylprednisolone represented the most commonly used agents, underscoring the central role of glucocorticoids in controlling inflammation in NPSLE. Immunosuppressive therapies were prescribed to 68.3% (82/120) of patients, with mycophenolate mofetil, cyclophosphamide, and methotrexate frequently employed, reflecting their central contribution to preventing disease exacerbation and managing severe organ involvement. Immunomodulators such as hydroxychloroquine and chloroquine were used in 37.5% (45/120) of cases, highlighting their sustained importance in long-term disease control.

**Figure 3:**
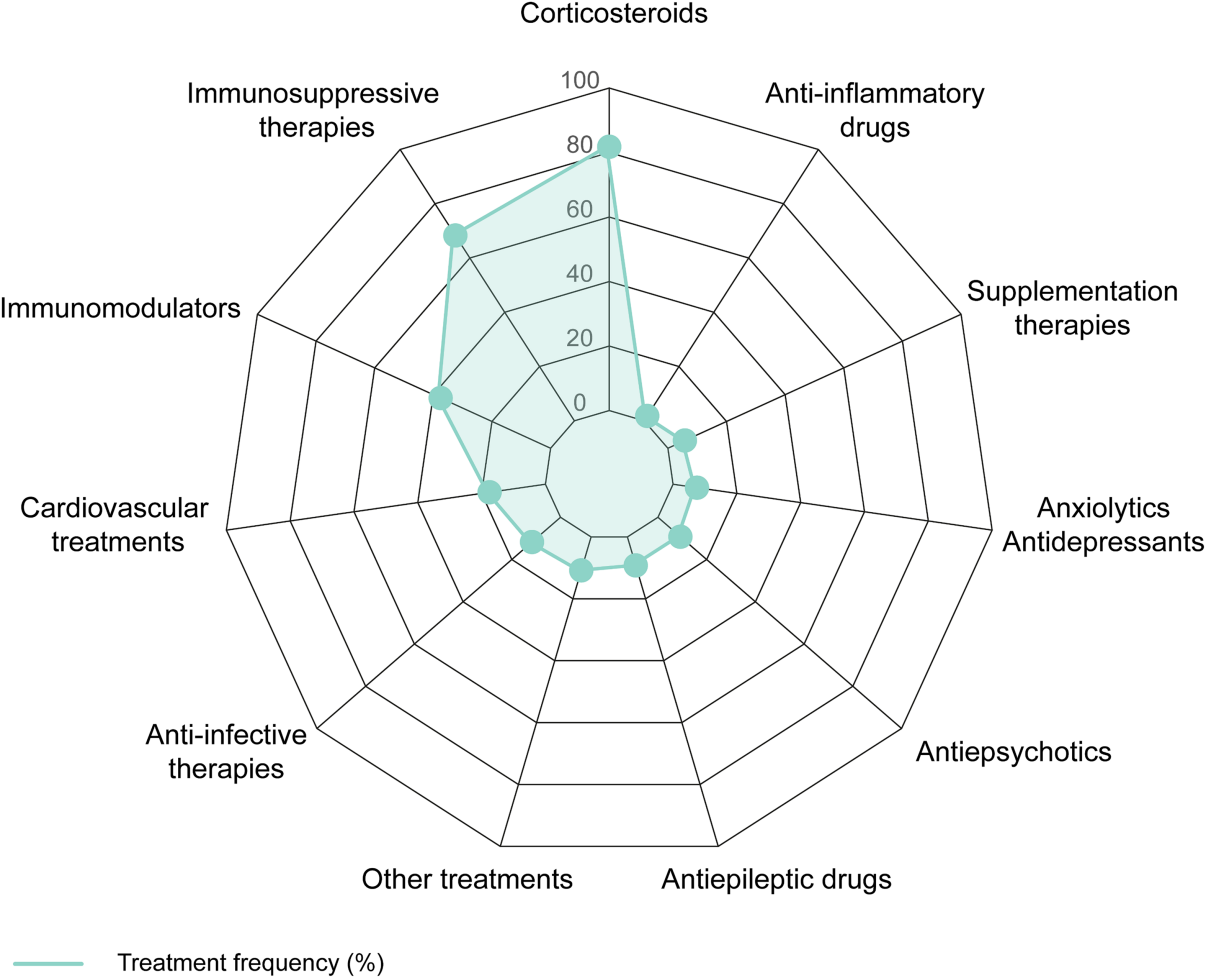
Distribution of treatments administered to NPSLE patients. Relative frequencies (%) of the main therapeutic classes reported across included cases, including corticosteroids, immunosuppressive agents, immunomodulators, cardiovascular treatments, anti-infective agents, neurological and psychotropic medications, anti-inflammatory drugs, supplementation therapies, and other treatments (e.g., plasmapheresis and immunoglobulins).

Cardiovascular treatments were recorded in 17.5% (21/120) of patients and addressed comorbidities such as thrombosis or hypertension, with agents including aspirin, warfarin, and amlodipine. Anti-infective therapies were prescribed in 11.7% (14/120) of cases, spanning antibiotics (e.g., ciprofloxacin, vancomycin), antivirals (acyclovir), and antifungals (posaconazole) (**Figure 3**). Antiepileptic drugs such as levetiracetam and valproic acid were prescribed to only 9.2% (11/120) of patients, despite seizures, convulsions, and consciousness disorders affecting nearly 60% of the cohort. Antipsychotics, including quetiapine and olanzapine, were administered at similar frequencies (9.2%), and anxiolytics or antidepressants (sertraline, diazepam, amitriptyline) were prescribed in only 7.5% (9/120) of patients. These rates contrast sharply with the high prevalence of mood and affective disorders (57.5%) and behavioral or psychotic disturbances (46.2%).

Other therapeutic categories were less frequently represented. Anti-inflammatory agents such as meloxicam and naproxen were used in only 1.7% (2/120) of patients, and supplementation therapies (calcium, vitamin D, iron) were noted in 5.8% (7/120). Additional interventions—including plasmapheresis, intravenous immunoglobulins, and neuropathic pain management with agents such as gabapentin—were recorded in 10.8% (13/120) of patients.

#### Treatment distribution by age and sex categories

The distribution of treatments across age and sex groups revealed distinct patterns, reflecting tailored therapeutic decisions in NPSLE management. Corticosteroids remained a foundational therapy in all subgroups, with the highest use observed in juvenile (89.3%, 25/28), followed by adult (79.3%, 65/82) and elderly patients (80%, 8/10), without evidence of a significant age-related trend (*P* = 0.32). Female patients were more frequently treated with corticosteroids (83.2%, 84/101) than male patients (73.7%, 14/19), although this difference did not reach statistical significance (*P* = 0.34). Immunosuppressive therapies were commonly prescribed, particularly in adult- (73.2%, 60/82) and male patients (73.7%, 14/19). Their use was lower in juvenile (53.6%, 15/28) and remained high in elderly patients (70%, 7/10), without a statistically significant age-related trend (*P* = 0.12). The higher prevalence observed in males compared with females did not reach statistical significance (*P* = 0.79). Immunomodulators such as hydroxychloroquine were predominantly used in adult (45.1%, 37/82) and in female patients (39.6%, 40/101). Usage was lower in male patients (26.3%, 5/19) and markedly reduced in elderly patients (10%, 1/10).

Cardiovascular therapies, including aspirin and warfarin, were more commonly prescribed in adult (19.5%, 16/82) than in juvenile (14.3%, 4/28) or elderly patients (10%, 1/10), without evidence of a significant age-related trend (*P* = 0.95). Female patients were more frequently treated (19.8%, 20/101) than male patients (5.3%, 1/19), although this difference did not reach statistical significance (*P* = 0.19). Anti-infective therapies were infrequently used overall. Adult patients showed the highest usage (14.6%, 12/82), followed by male (15.8%, 3/19) and female patients (10.9%, 11/101). Juvenile patients exhibited limited use (7.1%, 2/28), and no elderly patients received anti-infective agents, without evidence of a significant age-related trend (*P* = 0.96) or sex-based difference (*P* = 0.46).

Psychotropic medications revealed limited sex- and age-related differences. Anxiolytics and antidepressants were administered to 8.9% (9/101) of female patients, whereas none of the male patients received these therapies; however, this difference was not statistically significant (*P* = 0.35). Antipsychotics were used at comparable rates across age groups (juvenile 10.7%, 3/28; adult 8.5%, 7/82; elderly 10%, 1/10), without a significant age-related trend (*P* = 0.84). Supplementation therapies, including calcium, vitamin D, and iron, were reported primarily in juvenile (7.1%, 2/28) and in female patients (6.9%, 7/101). No supplementation was reported among male or elderly patients.

#### Outcome analysis

At the last recorded follow-up, most patients achieved a favorable clinical outcome, with 74.2% (89/120) showing improvement or stability, while 25.8% (31/120) experienced unfavorable outcomes, including deaths. Examination across age categories did not reveal a significant difference in outcome distribution. Adult patients showed a favorable outcome rate of 74.4% (61/82), whereas 25.6% (21/82) had unfavorable trajectories. Elderly patients exhibited a favorable outcome rate of 80% (8/10), with 20% (2/10) classified as unfavorable. Juvenile patients demonstrated a favorable outcome rate of 71.4% (20/28) and an unfavorable outcome rate of 28.6% (8/28), without evidence of a significant age-related trend (*P* = 0.60). Sex-based comparisons also showed similar outcome patterns. Favorable outcomes were observed in 78.9% (15/19) of male patients and in 73.3% (74/101) of female patients, with no statistically significant difference between sexes (*P* = 0.78).

#### Treatment efficacy on outcomes

To assess the impact of major therapeutic categories on clinical outcomes, an odds ratio (OR) analysis was performed using dichotomized endpoints (improvement = 1; no improvement or death = 0). Immunosuppressive therapies were more frequently reported among cases with favorable outcomes (OR 3.23, 95% CI 1.32–8.06; FDR-adjusted *P* = 0.016). Immunomodulators, including hydroxychloroquine, did not demonstrate a significant association with outcome (OR 0.79, 95% CI 0.30–2.13; FDR-adjusted *P* = 0.644), suggesting limited direct impact on short-term clinical improvement.

Corticosteroids exhibited the strongest association with favorable outcomes in this descriptive dataset (OR 4.04, 95% CI 1.40–12.08; FDR-adjusted *P* = 0.016). Patients receiving corticosteroids were approximately four times more likely to improve compared with those not treated with glucocorticoids, reinforcing their central role in mitigating inflammation and preventing deterioration in NPSLE.

## Discussion

**Figure 4:**
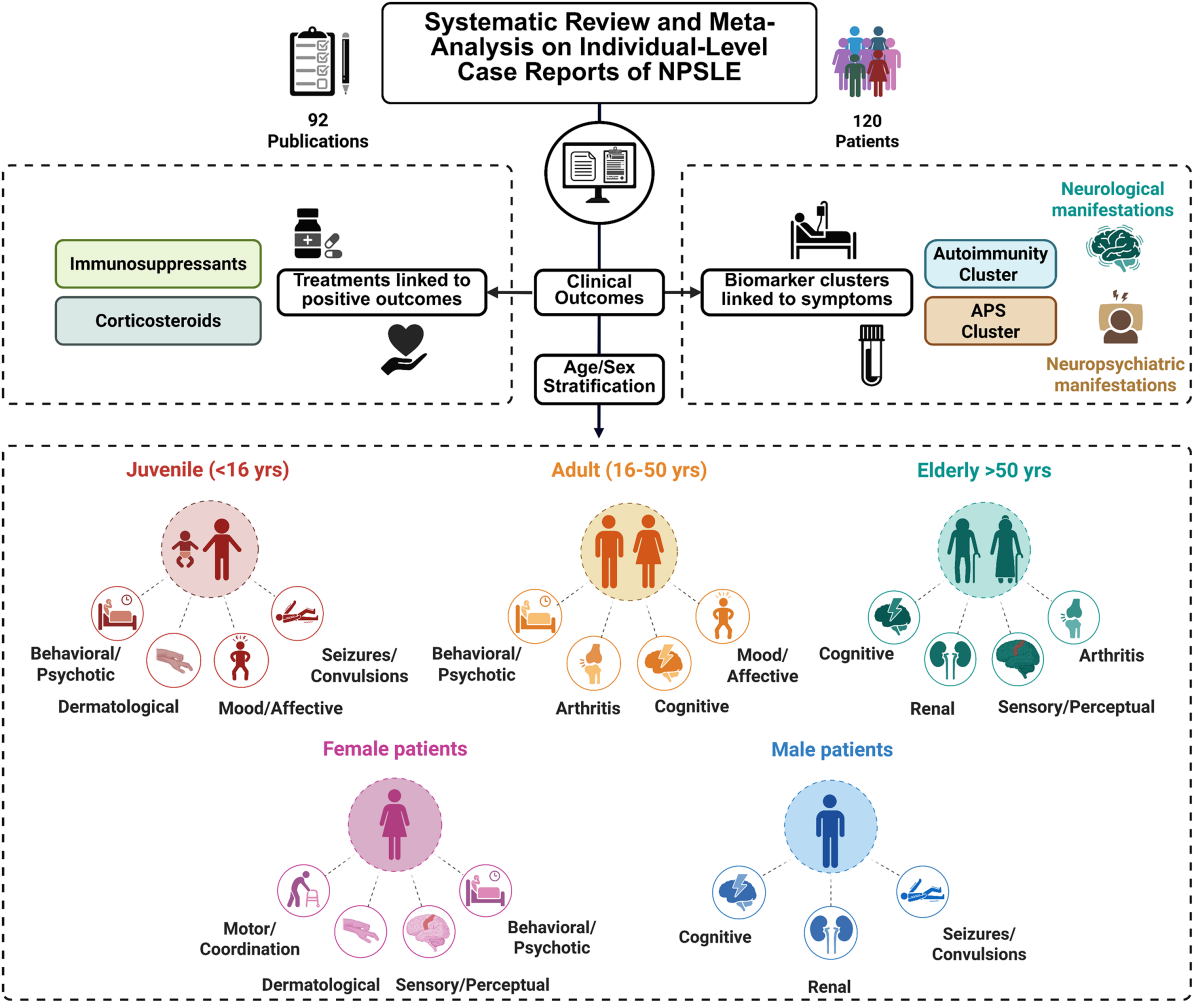
Graphical summary of the NPSLE systematic review. Schematic overview summarizing the main clinical characteristics, biomarker clusters, neuroimaging findings, and treatment categories identified across the included studies, stratified by age at presentation and sex.

Neuropsychiatric lupus (NPSLE) represents one of the most challenging and heterogeneous manifestations of SLE, with presentations ranging from subtle cognitive or affective changes to severe neurological events. In this systematic review integrating individual case reports, we delineated demographic patterns, biological signatures, neuroimaging findings, and therapeutic responses to better understand the mechanisms driving symptom variability and to support more personalized approaches to diagnosis and treatment (**Figure 4**).

Across the pooled dataset, neurological and neuropsychiatric manifestations were more prevalent in adult and elderly patients, consistent with prior reports indicating that cognitive dysfunction, seizures, and cerebrovascular complications are particularly difficult to diagnose and treat in older patients due to overlapping comorbidities and atypical symptom evolution (111, 112). In contrast, juvenile patients more frequently exhibited dermatological manifestations and behavioral or psychotic disturbances, a pattern potentially influenced by immune system maturation, hormonal, or age-dependent genetic factors shaping tissue-specific vulnerability (113–115). The higher rate of renal involvement at the extremes of age, as observed here and in previous cohorts (116), reinforces the need for vigilant renal monitoring in pediatric and older patients (**Figure 4**).

Importantly, normal aging is associated with progressive structural and biochemical brain changes that increase vulnerability to cognitive and neuropsychiatric symptoms, even in healthy individuals. Age therefore represents a potential confounding factor in the interpretation of neurological and neuropsychiatric severity in NPSLE, particularly in adult- and elderly cases. In this context, worsened outcomes in older patients should be interpreted as the result of cumulative interactions between age-related brain vulnerability and chronic autoimmune-mediated injury, rather than disease-specific neurodegeneration alone. This underscores the need for future studies to differentiate age-related brain vulnerability from disease-specific injury using age-matched controls and standardized neuropsychological and imaging assessments.

Sex-specific trends were also evident. Although SLE predominantly affects women, male patients in our dataset more frequently exhibited renal involvement, in alignment with registry data showing that SLE in males, though less common, is often clinically aggressive with severe organ manifestations (117). Female patients, conversely, displayed higher rates of dermatological and neuropsychiatric symptoms, including cognitive impairment and mood disturbances. These differences may reflect immunohormonal influences, particularly estrogen-mediated modulation of immune activation (115) and highlight the importance of sex-adapted screening strategies, especially for neuropsychiatric evaluation (**Figure 4**).

To disentangle the complexity of biological markers associated with NPSLE, we applied principal component analysis and identified four biomarker clusters. The “*Autoimmunity and Inflammation*” cluster, encompassing anti-dsDNA, reduced complement, and related autoantibodies, showed the strongest association with neurological symptoms. This reinforces the well-established concept that BBB disruption facilitates the entry of neurotoxic antibodies such as anti-NMDA receptor and anti-dsDNA into the CNS, triggering neuroinflammation and neuronal injury (2, 118). The correlation of these markers with neurological dysfunction echoes emerging evidence implicating microglial activation in NPSLE neuropathology (119) and is consistent with lupus-like murine models demonstrating apoptosis, synaptic dysfunction, and BBB breakdown following autoantibody deposition (120, 121). Newer biomarkers, including suprabasin autoantibodies and CSF neurofilament light chain, may further improve diagnostic accuracy and help distinguish NPSLE from other SLE phenotypes (1, 122), although validation in larger cohorts is required.

The “*Kidney and Metabolic Dysfunction*” cluster was specifically associated with renal manifestations, consistent with long-term registry data identifying nephritis as one of the most clinically significant SLE complications (123). The observation that many patients displayed substantial proteinuria despite preserved electrolyte profiles and normal creatinine levels suggests diverse renal injury trajectories, potentially reflecting varying degrees of glomerular versus tubular involvement. Moderate correlations between renal biomarkers and certain immunological parameters (e.g., anti-SSA, anti-SSB) point to shared autoimmune pathways capable of simultaneously affecting the kidney and CNS.

The “*Thrombosis and Antiphospholipid Syndrome*” cluster showed a borderline association with neuropsychiatric symptoms, consistent with the concept of “lupus vasculopathy,” characterized by microthrombosis, endothelial dysfunction, and complement activation (124, 125). Antiphospholipid-mediated microangiopathy has been linked to silent infarctions, microbleeds, and subclinical cognitive deficits, providing a plausible mechanistic bridge between autoimmunity and neurovascular injury (126, 127). In contrast, the “*Hematology and Immune Cells*” cluster showed no specific association with symptom categories, suggesting more global rather than phenotype-specific hematological perturbations.

Neuroimaging analysis confirmed MRI as the most sensitive modality for detecting neurostructural abnormalities, identifying lesions in more than 80% of patients in whom neuroimaging was performed. Common findings, including white matter hyperintensities, cortical atrophy, and focal lesions in deep gray matter nuclei, align with previous radiological descriptions of NPSLE (128–130). MRI abnormalities correlated most strongly with motor, sensory, and coordination deficits, supporting the involvement of inflammatory and ischemic processes in pathways governing these functions (129, 131). Despite its utility, standard MRI remains limited by its nonspecific findings and the lack of harmonized protocols. Advanced imaging modalities—including diffusion tensor imaging, functional MRI, quantitative susceptibility mapping, and dynamic contrast-enhanced MRI—have demonstrated promise in detecting microstructural abnormalities, altered connectivity, microhemorrhages, and BBB permeability (132–134). PET imaging with TSPO ligands further offers a window into CNS inflammatory activity (135–137). While these technologies are not yet widely adopted, strategic use in high-risk or diagnostically ambiguous cases may enhance clinical decision-making.

Therapeutically, corticosteroids (81.7%) and immunosuppressants (68.3%) were the most frequently used agents, and logistic regression indicated significant associations between these treatments and favorable outcomes. While these associations reflect treatment patterns reported in published cases and should not be interpreted as evidence of comparative therapeutic efficacy, these findings reinforce their central role in controlling inflammation and preventing irreversible organ damage. However, concerns remain regarding optimal dosing, long-term toxicity, and their impact on specific neuropsychiatric endpoints (138–140). Notably, psychotropic medications and antiepileptic agents were markedly underutilized compared with the prevalence of mood disturbances, cognitive deficits, and seizures. This discrepancy may reflect under-recognition of neuropsychiatric symptoms, reluctance to escalate pharmacotherapy due to polypharmacy concerns, or a lack of robust evidence guiding psychotropic and antiepileptic use in NPSLE (125). Implementing standardized neuropsychiatric assessment tools in routine care could improve detection and inform earlier, more targeted interventions.

Biologic therapies such as belimumab and anifrolumab have shown efficacy in reducing systemic SLE activity (141), and early reports suggest potential benefit for neuropsychiatric manifestations (142, 143). Their ability to modulate autoreactive B-cell activity and type I interferon signaling positions them as promising adjuncts, especially in refractory cases. Combining biologics with therapies targeting BBB permeability or microglial activation, plasmapheresis, intravenous immunoglobulins, or experimental small molecules, represents a future therapeutic frontier (144). Multicenter randomized trials incorporating neuropsychiatric endpoints are needed to clarify the role of these agents in NPSLE.

Taken together, converging evidence across clinical, biological, and imaging domains highlights the mechanistic complexity of NPSLE, encompassing autoantibody-mediated neuronal injury, microvascular dysfunction, complement activation, and inflammatory cascades. This complexity underscores the need for biomarker-driven patient stratification to better capture disease heterogeneity. In this context, integrated biomarker profiling primarily serves to structure heterogeneous clinical presentations and to generate testable hypotheses, rather than to establish mechanistic causality. Our cluster-based approach may help structure clinical heterogeneity and generate hypotheses supporting the development of more personalized immunomodulatory strategies. Translational models further support this concept, demonstrating that selective targeting of autoantibody subsets or inflammatory mediators can confer organ-specific protection (138, 139). The clinical implementation of these precision approaches will depend on robust biomarker validation and an improved understanding of longitudinal disease trajectories.

## Limitations

This systematic review has several limitations inherent to retrospective, case-report–based evidence. Published case reports preferentially describe severe, atypical, or diagnostically challenging presentations, which may limit the generalizability of the findings to the broader NPSLE population. Consequently, reported frequencies and associations should be interpreted as descriptive rather than representative estimates. Treatment–outcome relationships are particularly subject to confounding by indication and reporting bias, as therapeutic decisions are influenced by disease severity, clinical context, and physician judgment. As such, treatment-associated outcomes observed in this synthesis cannot be interpreted as evidence of efficacy or comparative benefit. Biological data were frequently incomplete across reports, necessitating imputation for multivariate analyses. Although this approach preserved the overall data structure, missingness mechanisms could not be formally assessed and may influence exploratory clustering results. In addition, substantial heterogeneity in imaging protocols, laboratory assays, reporting standards, and the exclusion of non-English studies limit cross-study comparability. Finally, the observational and retrospective nature of the included evidence precludes causal inference. Despite these limitations, this systematic synthesis provides a structured framework to generate testable hypotheses and underscores the need for prospective multicenter cohorts using standardized neuropsychiatric assessments, integrated biomarker panels, and harmonized imaging protocols. Future research should also prioritize randomized trials evaluating biologics and neuroprotective strategies, while leveraging insights from experimental models of BBB dysfunction and microglial activation to inform targeted therapeutic development (1, 145, 146).

## Data Availability

The original contributions presented in the study are included in the article/**Supplementary Material**. Further inquiries can be directed to the corresponding authors.

## Supplementary material

Supplementary Figure 1 PRISMA (Preferred Reporting Items for Systematic Reviews and Meta-Analyses) flowchart for Individual Participant Data (IPD).

Supplementary Figure 2 Correlation matrix of biological, hematological, and immunological parameters in NPSLE.

Supplementary Figure 3 Principal Component Analysis (PCA) of biomarkers showing four clusters based on shared variance: Autoimmunity and Inflammation, Kidney and Metabolic Dysfunction, Thrombosis and Antiphospholipid Syndrome, and Hematology and Immune Cells.
