# Supplementary material for: A multidimensional analysis of neuropsychiatric lupus: clinical, biological and imaging insights from systematic evidence
Source: Front Immunol. 2026 Mar 16;17:1768131. doi: 10.3389/fimmu.2026.1768131 (PMC13033608; doi:10.3389/fimmu.2026.1768131)
Supplement: Supplementary file 1 [file Table1.docx]

**Supplementary Table 1: Epidemiologic and clinical characteristics of patients diagnosed with NPSLE.**

|  | | Number of patients | Age at diagnosis* | Age at last  exam* | Skin Rash and related symptoms,  n (%) | Arthritis and related symptoms,  n (%) | Sign of renal involvement,  n (%) | Splenomegaly, n (%) | Neurological symptoms,  n (%**) | Neuropsychiatric symptoms,  n (%**) | Brain MRI abnormalities, n (%***) | Brain CT scan abnormalities,  n (%***) | Brain CT angiography abnormalities,  n (%***) | CSF abnormalities,  n (%***) |
| --- | --- | --- | --- | --- | --- | --- | --- | --- | --- | --- | --- | --- | --- | --- |
| All patients | All ages | 120 | 22.5  [16-36.25] | 28.5  [20-41.25] | 58  (48.3%) | 56  (46.7%) | 44  (36.6%) | 9  (7.5%) | 106  (88.3%) | 80  (66.7%) | 80 (81.6%) | 22 (55.0%) | 5 (50.0%) | 46 (59.7%) |
|  | Juvenile-onset  (<16 yrs) | 28 | 11  [4.8-14] | 15.35  [8.25-20] | 18  (64.3%) | 8  (28.6%) | 13  (46.4%) | 4  (14.3%) | 22  (78.6%) | 14  (50.0%) | 14  (82.3%) | 3 (75.0%) | 1 (50.0%) | 4 (40.0%) |
|  | Adult-onset  (16-50 yrs) | 82 | 25.5  [21-35.75] | 30.25  [24-40.75] | 35  (42.7%) | 42  (51.2%) | 25  (30.5%) | 5  (6.1%) | 74  (90.2%) | 58  (70.7%) | 57 (79.2%) | 17 (53.1%) | 4 (50.0%) | 38 (63.3%) |
|  | Elderly-onset  (>50 yrs) | 10 | 61.5  [55.75-67] | 61.5  [58.25-67] | 5  (50.0%) | 6  (60.0%) | 6  (60.0%) | 0  (0.0%) | 10  (100.0%) | 8  (80.0%) | 9 (100.0%) | 2 (50.0%) | 0 (0.0%) | 4 (57.1%) |
| Females | All ages | 101 | 23  [16-35] | 29  [21-41] | 52  (51.5%) | 48  (47.5%) | 34  (33.7%) | 3  (3.0%) | 88  (87.1%) | 68  (67.3%) | 67 (79.8%) | 21 (60.0%) | 4 (50.0%) | 38  (59.4%) |
|  | Juvenile-onset  (<16 yrs) | 22 | 13  [8.25-14.75] | 16.5  [12.62-20] | 15  (68.2%) | 7  (31.8%) | 9  (40.9%) | 0  (0.0%) | 17  (77.3%) | 11  (50.0%) | 12 (80.0%) | 3 (75.0%) | 0 (0.0%) | 4 (44.4%) |
|  | Adult-onset  (16-50 yrs) | 70 | 25  [21-34.75] | 30  [24-39.75] | 33  (47.1%) | 36  (51.4%) | 20  (28.6%) | 3  (4.3%) | 62  (88.6%) | 50  (71.4%) | 47 (77.0%) | 16 (59.3%) | 4 (57.1%) | 31 (63.3%) |
|  | Elderly-onset  (>50 yrs) | 9 | 64  [55-68] | 64  [58-68] | 4  (44.4%) | 5  (55.6%) | 5  (55.6%) | 0  (0.0%) | 9  (100.0%) | 7  (77.8%) | 8 (100.0%) | 2 (50.0%) | 0 (0.0%) | 3 (50.0%) |
| Male | All ages | 19 | 22  [9.5-38] | 24  [13.5-42] | 6  (31.6%) | 8  (42.1%) | 10  (52.6%) | 6  (31.6%) | 18  (94.7%) | 12  (63.2%) | 13 (92.9%) | 1 (20.0%) | 1 (50.0%) | 8 (61.5%) |
|  | Juvenile-onset  (<16 yrs) | 6 | 4.5  [3.37-8] | 7.5  [6-9.75] | 3  (50.0%) | 1  (16.7%) | 4  (66.7%) | 4  (66.7%) | 5  (83.3%) | 3  (50.0%) | 2 (100.0%) | 0 (0.0%) | 1 (100.0%) | 0 (0.0%) |
|  | Adult-onset  (16-50 yrs) | 12 | 34  [21.5-40] | 36.25  [23.75-45] | 2  (16.7%) | 6  (50.0%) | 5  (41.7%) | 2  (16.7%) | 12  (100.0%) | 8  (66.7%) | 10 (90.9%) | 1 (20.0%) | 0 (0.0%) | 7 (63.6%) |
|  | Elderly-onset  (>50 yrs) | 1 | 58  [58] | 59  [59] | 1  (100.0%) | 1  (100.0%) | 1  (100.0%) | 0  (0.0%) | 1  (100.0%) ​ | 1  (100.0%) | 1 (100.0%) | 0 (0.0%) ​ | 0 (0.0%) | 1 (100.0%) |

* Median (Years) [25th–75th interquartile range]

** Over the number of patients with reported neurological symptoms, comprising “*Motor, Vesicosphincterian and Coordination disorders*”, “*Sensory and Perceptual disorders*” ; “*Seizures/convulsions and Consciousness disorders*”.

** Over the number of patients with reported neuropsychiatric symptoms, including “*Cognitive disorders*” ; “*Mood and Affective disorders*” and “*Behavioral and Psychotic disorders*”.

*** Over the number of patients with imaging (MRI, CT, angiography) and CSF reported data.

*Abbreviations: CSF: Cerebrospinal Fluid; CT: Computed Tomography; MRI: Magnetic Resonance Imaging; n: number; NPSLE: Neuropsychiatric Systemic Lupus Erythematosus; yrs: years.*
