# Supplementary material for: A multidimensional analysis of neuropsychiatric lupus: clinical, biological and imaging insights from systematic evidence
Source: Front Immunol. 2026 Mar 16;17:1768131. doi: 10.3389/fimmu.2026.1768131 (PMC13033608; doi:10.3389/fimmu.2026.1768131)
Supplement: Supplementary file 2 [file Table2.docx]

| **Parameters** | **All patients, n (%*)** | | | | | **Juvenile-Onset, n (%*)** | | | | | **Adult-Onset, n (%*)** | | | | | **Elderly-Onset, n (%*)** | | | | |
| --- | --- | --- | --- | --- | --- | --- | --- | --- | --- | --- | --- | --- | --- | --- | --- | --- | --- | --- | --- | --- |
|  | **Normal** | **Low** | **High** | **Positive (+)** | **Negative**  **(-)** | **Normal** | **Low** | **High** | **Positive (+)** | **Negative**  **(-)** | **Normal** | **Low** | **High** | **Positive (+)** | **Negative**  **(-)** | **Normal** | **Low** | **High** | **Positive (+)** | **Negative**  **(-)** |
| Hemoglobin | 35 (38.9%) | 55 (61.1%) | 0 (0%) | N/A | N/A | 10 (43.5%) | 13 (56.2%) | 0 (0.0%) | N/A | N/A | 22 (37.3%) | 37 (62.7%) | 0 (0.0%) | N/A | N/A | 3 (37.5%) | 5 (62.5%) | 0 (0.0%) | N/A | N/A |
| MCV | 53 (89.8%) | 5 (8.5%) | 1 (1.7%) | N/A | N/A | 13 (86.7%) | 2 (13.3%) | 0 (0.0%) | N/A | N/A | 36 (90.0%) | 3 (7.5%) | 1 (2.5%) | N/A | N/A | 4 (100.0%) | 0 (0.0%) | 0 (0.0%) | N/A | N/A |
| Anti-cardiolipin IgM | N/A | N/A | N/A | 32 (37.6%) | 53 (62.3%) | N/A | N/A | N/A | 9 (40.9%) | 13 (59.1%) | N/A | N/A | N/A | 19 (33.3%) | 38 (66.7%) | N/A | N/A | N/A | 4 (66.7%) | 2 (33.3%) |
| Anti-cardiolipin IgG | N/A | N/A | N/A | 29 (32.6%) | 60 (67.4%) | N/A | N/A | N/A | 9 (40.9%) | 13 (59.1%) | N/A | N/A | N/A | 17 (28.3%) | 43 (71.7%) | N/A | N/A | N/A | 3 (42.9%) | 4 (57.1%) |
| WBC | 48 (51.6%) | 40 (43.0%) | 5 (5.4%) | N/A | N/A | 11 (44.0%) | 13 (52.0%) | 1 (4.0%) | N/A | N/A | 35 (57.4%) | 22 (36.1%) | 4 (6.6%) | N/A | N/A | 2 (28.6%) | 5 (71.4%) | 0 (0.0%) | N/A | N/A |
| Lymphocytes | 40 (51.9%) | 32 (41.6%) | 5 (6.5%) | N/A | N/A | 14 (58.3%) | 8 (33.3%) | 2 (8.3%) | N/A | N/A | 25 (53.2%) | 19 (40.4%) | 3 (6.4%) | N/A | N/A | 1 (16.7%) | 5 (83.3%) | 0 (0.0%) | N/A | N/A |
| Platelets | 51 (60.0%) | 34 (40.0%) | 0 (0.0%) | N/A | N/A | 10 (43.5%) | 13 (56.5%) | 0 (0.0%) | N/A | N/A | 39 (70.9%) | 16 (29.1%) | 0 (0.0%) | N/A | N/A | 2 (28.6%) | 5 (71.4%) | 0 (0.0%) | N/A | N/A |
| CRP | 24 (28.2%) | N/A | 61 (71.8%) | N/A | N/A | 8 (34.8%) | N/A | 15 (65.2%) | N/A | N/A | 15 (27.8%) | N/A | 39 (72.2%) | N/A | N/A | 0 (0.0%) | 1 (12.5%) | 7 (87.5%) | N/A | N/A |
| Sedimentation Rate | 6 (8.1%) | N/A | 68 (91.9%) | N/A | N/A | 2 (12.5%) | N/A | 14 (87.5%) | N/A | N/A | 4 (8.0%) | N/A | 46 (92.0%) | N/A | N/A | 0 (0.0%) | 0 (0.0%) | 8 (100.0%) | N/A | N/A |
| Bilirubin | 63 (86.3%) | 0 (0.0%) | 10 (13.7%) | N/A | N/A | 16 (80.0%) | 0 (0.0%) | 4 (20.0%) | N/A | N/A | 42 (89.4%) | 0 (0.0%) | 5 (10.6%) | N/A | N/A | 5 (83.3%) | 0 (0.0%) | 1 (16.7%) | N/A | N/A |
| Urea, Plasma (BUN) | 64 (77.1%) | 2 (2.4%) | 17 (20.5%) | N/A | N/A | 15 (62.5%) | 1 (4.2%) | 8 (33.3%) | N/A | N/A | 42 (82.3%) | 1 (2.0%) | 8 (15.7%) | N/A | N/A | 7 (87.5%) | 0 (0.0%) | 1 (12.5%) | N/A | N/A |
| Creatinine | 74 (83.1%) | 2 (2.2%) | 13 (14.6%) | N/A | N/A | 17 (68%) | 0 (0.0%) | 8 (32.0%) | N/A | N/A | 50 (89.3%) | 2 (3.6%) | 4 (7.1%) | N/A | N/A | 7 (87.5%) | 0 (0.0%) | 1 (12.5%) | N/A | N/A |
| Sodium | 59 (85.5%) | 10 (14.5%) | 0 (0.0%) | N/A | N/A | 14 (77.8%) | 4 (22.2%) | 0 (0.0%) | N/A | N/A | 38 (88.4%) | 5 (11.6%) | 0 (0.0%) | N/A | N/A | 7 (87.5%) | 1 (12.5%) | 0 (0.0%) | N/A | N/A |
| Potassium | 59 (88.1%) | 6 (9.0%) | 2 (3.0%) | N/A | N/A | 13 (76.5%) | 4 (23.5%) | 0 (0.0%) | N/A | N/A | 39 (90.8%) | 2 (4.6%) | 2 (4.6%) | N/A | N/A | 7 (100.0%) | 0 (0.0%) | 0 (0.0%) | N/A | N/A |
| Chloride | 58 (92.1%) | 1 (1.6%) | 4 (6.3%) | N/A | N/A | 13 (76.5%) | 0 (0.0%) | 4 (23.5%) | N/A | N/A | 40 (97.6%) | 1 (2.4%) | 0 (0.0%) | N/A | N/A | 5 (100.0%) | 0 (0.0%) | 0 (0.0%) | N/A | N/A |
| C3 | 20 (20.0%) | 77 (77.0%) | 3 (3.0%) | N/A | N/A | 13 (52.0%) | 11 (44.0%) | 1 (4.0%) | N/A | N/A | 7 (10.3%) | 59 (86.8%) | 2 (2.9%) | N/A | N/A | 0 (0.0%) | 7 (100.0%) | 0 (0.0%) | N/A | N/A |
| C4 | 19 (20.21%) | 75 (79.8%) | 0 (0.0%) | N/A | N/A | 12 (50.0%) | 12 (50.0%) | 0 (0.0%) | N/A | N/A | 0 (0.0%) | 57 (89.1 %) | 7 (10.9%) | N/A | N/A | 0 (0.0%) | 6 (100.0%) | 0 (0.0%) | N/A | N/A |
| ANAs | N/A | N/A | N/A | 118 (99.2%) | 1 (0.8%) | N/A | N/A | N/A | 27 (96.4%) | 1 (3.6%) | N/A | N/A | N/A | 81 (100.0%) | 0 (0.0%) | N/A | N/A | N/A | 10 (100.0%) | 0 (0.0%) |
| Anti-dsDNA | N/A | N/A | N/A | 93 (84.5%) | 17 (15.4%) | N/A | N/A | N/A | 16 (61.5%) | 10 (38.5%) | N/A | N/A | N/A | 69 (93.2%) | 5 (6.8%) | N/A | N/A | N/A | 8 (80.0%) | 2 (20.0%) |
| Anti-SSA | N/A | N/A | N/A | 47 (77.0%) | 14 (22.9%) | N/A | N/A | N/A | 10 (83.3%) | 2 (16.7%) | N/A | N/A | N/A | 34 (77.3%) | 10 (22.7%) | N/A | N/A | N/A | 3 (60.0%) | 2 (40.0%) |
| Anti-SSB | N/A | N/A | N/A | 26 (50.0%) | 26 (50.0%) | N/A | N/A | N/A | 9 (81.8%) | 2 (18.2%) | N/A | N/A | N/A | 15 (41.7%) | 21 (58.3%) | N/A | N/A | N/A | 2 (40.0%) | 3 (60.0%) |
| Anti-RNP | N/A | N/A | N/A | 36 (69.2%) | 16 (30.8%) | N/A | N/A | N/A | 10 (83.3%) | 2 (16.7%) | N/A | N/A | N/A | 25 (69.4%) | 11 (30.6%) | N/A | N/A | N/A | 1 (25.0%) | 3 (75.0%) |
| Anti-Sm | N/A | N/A | N/A | 38 (74.5%) | 13 (25.5%) | N/A | N/A | N/A | 8 (88.9%) | 1 (11.1%) | N/A | N/A | N/A | 27 (71.1%) | 11 (28.9%) | N/A | N/A | N/A | 3 (75.0%) | 1 (25.0%) |
| Anti-beta 2-glycoprotein I | N/A | N/A | N/A | 19 (35.2%) | 35 (64.8%) | N/A | N/A | N/A | 9 (60.0%) | 6 (40.0%) | N/A | N/A | N/A | 10 (28.6%) | 25 (71.4%) | N/A | N/A | N/A | 0 (0.0%) | 4 (100.0%) |
| Lupus Anticoagulant | N/A | N/A | N/A | 24 (33.8%) | 47 (66.2%) | N/A | N/A | N/A | 10 (58.8%) | 7 (41.2%) | N/A | N/A | N/A | 12 (25.0%) | 36 (75.0%) | N/A | N/A | N/A | 2 (33.3%) | 4 (66.7%) |
| Proteinuria | N/A | N/A | N/A | 56 (84.8%) | 10 (15.1%) | N/A | N/A | N/A | 15 (75.0%) | 5 (25.0%) | N/A | N/A | N/A | 34 (87.2%) | 5 (12.8%) | N/A | N/A | N/A | 7 (100.0%) | 0 (0.0%) |

**Supplementary Table 2: Biological and biochemical analysis of diagnosed patients.**

*Over the number of total patients with reported data.

*Abbreviations: (+/-): positive/negative test; ANAs: Antinuclear Antibodies; Anti-dsDNA: Anti-double stranded DNA; Anti-RNP: Anti-Ribonucleoprotein; Anti-Sm: Anti-Smith Antibody; Anti-SSA: Anti-Sjögren's syndrome-related antigen A; Anti-SSB: Anti-Sjögren's syndrome-related antigen B; BUN: Blood urea nitrogen; C3: Complement Component 3; C4: Complement Component 4; CNS: Central Nervous System; CRP: C-Reactive Protein; IgG: Immunoglobulin G; IgM: Immunoglobulin M; MCV: Mean Corpuscular Volume; N/A: Not Applicable; NPSLE: neuropsychiatric Systemic Lupus Erythematosus; n: number; RBC: Red Blood Cells;WBC: White Blood Cells.*
