# Supplementary material for: A multidimensional analysis of neuropsychiatric lupus: clinical, biological and imaging insights from systematic evidence
Source: Front Immunol. 2026 Mar 16;17:1768131. doi: 10.3389/fimmu.2026.1768131 (PMC13033608; doi:10.3389/fimmu.2026.1768131)
Supplement: Supplementary file 3 [file Table3.docx]

**Supplementary Table 3: Neurological and neuropsychiatric symptoms described in patients diagnosed with NPSLE.**

|  | | Motor, vesicosphincterian and coordination disorders  n (%*) | Sensory and perceptual disorders  n (%*) | Seizures, convulsions, and consciousness disorders  n (%*) | Cognitive disorders  n (%*) | Mood and affective disorders  n (%*) | Behavioral and psychotic disorders  n (%*) |
| --- | --- | --- | --- | --- | --- | --- | --- |
| All patients | All ages | 58/106  (54.7%) | 52/106  (49.1%) | 63/106  (59.4%) | 62/80 (77.5%) | 46/80 (57.5%) | 37/80 (46.2%) |
|  | Juvenile-onset  (< 16 yrs) | 12/22  (54.5%) | 4/22  (18.2%) | 16/22  (72.7%) | 9/14 (64.3%) | 7/14 (50.0%) | 7/14 (50.0%) |
|  | Adulte-onset  (16-50 yrs) | 39/73  (53.4%) | 43/73  (58.9%) | 40/73  (54.8%) | 44/57 (77.2%) | 36/57 (63.2%) | 27/57 (47.4%) |
|  | Elderly-onset  (> 50 yrs) | 7/11  (63.6%) | 5/11  (45.4%) | 7/11  (63.6%) | 9/9 (100.0%) | 3/9 (33.3%) | 3/9 (33.3%) |
| Females | All ages | 52/88 (59.1%) | 45/88 (51.1%) | 50/88 (56.8%) | 52/68 (76.5%) | 40/68 (58.8%) | 30/68 (44.1%) |
|  | Juvenile-onset  (< 16 yrs) | 11/17  (64.7%) | 4/17 (23.5%) | 11/17 (64.7%) | 7/11 (63.6%) | 7/11 (63.6%) | 5/11 (45.4%) |
|  | Adult-onset  (16-50 yrs) | 35/62 (56.4%) | 37/62 (59.7%) | 34/62 (54.8%) | 38/50 (76.0%) | 31/50 (62.0%) | 23/50 (46.0%) |
|  | Elderly-onset  (> 50 yrs) | 6/9 (66.7%) | 4/9 (44.4%) | 5/9 (55.6%) | 7/7 (100.0%) | 2/7 (28.6%) | 2/7 (28.6%) |
| Males | All ages | 6/18 (33.3%) | 7/18 (38.9%) | 13/18 (72.2%) | 10/12 (83.3%) | 6/12 (50.0%) | 7/12 (58.3%) |
|  | Juvenile-onset  (< 16 yrs) | 1/5 (20.0%) | 0/5 (0.0%) | 5/5 (100.0%) | 2/3 (66.7%) | 0/3 (0.0%) | 2/3 (66.7%) |
|  | Adult-onset  (16-50 yrs) | 4/11 (36.4%) | 6/11 (54.5%) | 6/11 (54.55%) | 6/7 (85.7%) | 5/7 (71.4%) | 4/7 (57.1%) |
|  | Elderly-onset  (> 50 yrs) | 1/2 (50.0%) | 1/2 (50.0%) | 2/2 (100.0%) | 2/2 (100.0%) | 1/2 (50.0%) | 1/2 (50.0%) |

* Over the number of total patients with neurological symptoms.

*Abbreviations: n: number; yrs: years.*
