# Supplementary figures and images for: A multidimensional analysis of neuropsychiatric lupus: clinical, biological and imaging insights from systematic evidence
Source: Front Immunol. 2026 Mar 16;17:1768131. doi: 10.3389/fimmu.2026.1768131 (PMC13033608; doi:10.3389/fimmu.2026.1768131)

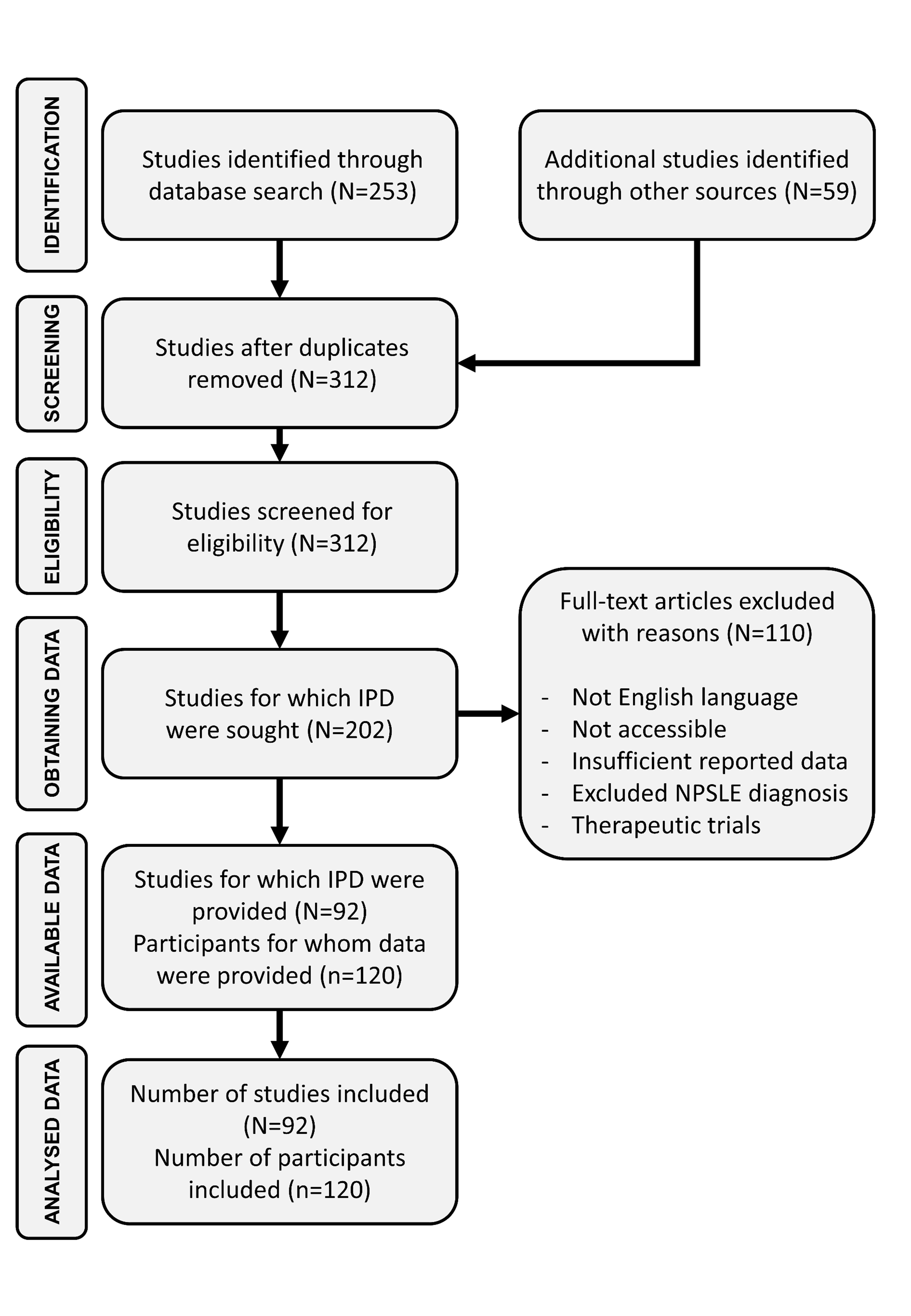

Supplement: Supplementary Figure 1 — PRISMA (Preferred Reporting Items for Systematic Reviews and Meta-Analyses) flowchart for Individual Participant Data (IPD). [file Image1.tif]

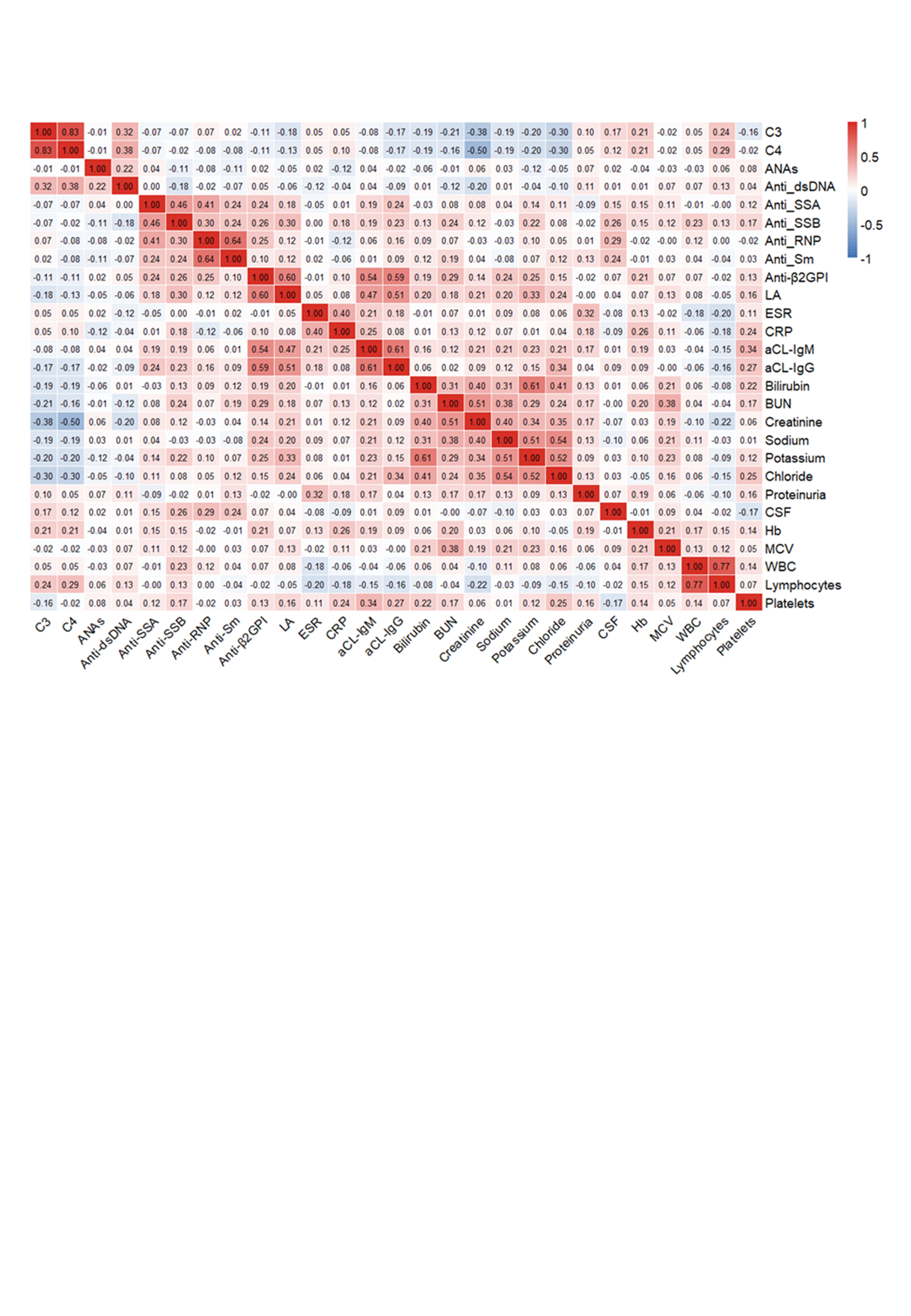

Supplement: Supplementary Figure 2 — Correlation matrix of biological, hematological, and immunological parameters in NPSLE. [file Image2.tif]

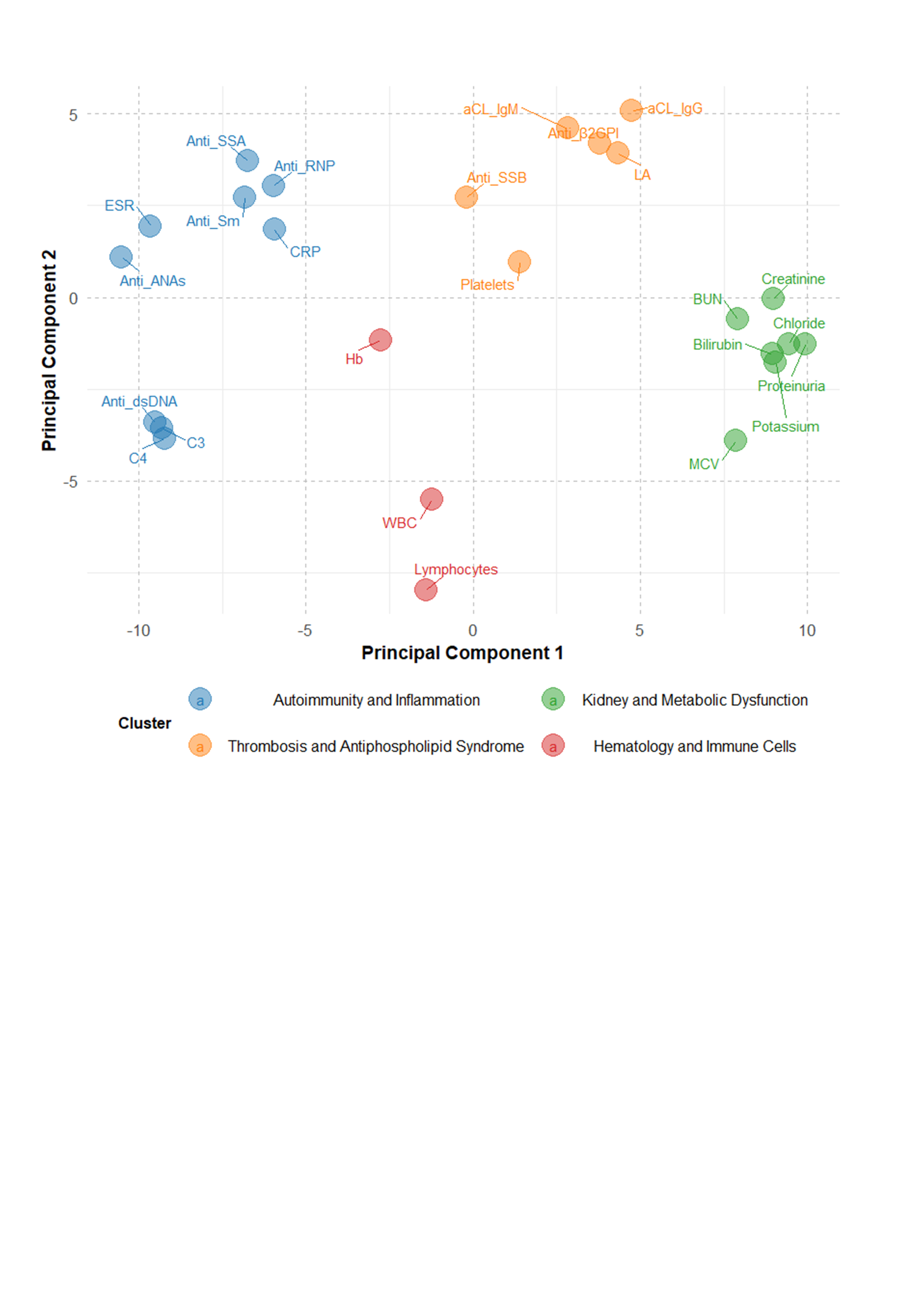

Supplement: Supplementary Figure 3 — Principal Component Analysis (PCA) of biomarkers showing four clusters based on shared variance: Autoimmunity and Inflammation, Kidney and Metabolic Dysfunction, Thrombosis and Antiphospholipid Syndrome, and Hematology and Immune Cells. [file Image3.tif]
